# Pipeline for Targeted Meta-Proteomic Analyses to Assess the Diversity of Cattle Rumen Microbial Urease

**DOI:** 10.3389/fmicb.2020.573414

**Published:** 2020-09-18

**Authors:** Xiaoyin Zhang, Shengguo Zhao, Yue He, Nan Zheng, Xianghua Yan, Jiaqi Wang

**Affiliations:** ^1^College of Animal Science and Technology, Huazhong Agricultural University, Wuhan, China; ^2^State Key Laboratory of Animal Nutrition, Institute of Animal Sciences, Chinese Academy of Agricultural Sciences, Beijing, China

**Keywords:** urease, trypsin, Glu-C/Lys-C, in-gel, in-solution, meta-proteomics

## Abstract

In the rumen of cattle, urease produced by ureolytic bacteria catalyzes the hydrolysis of urea to ammonia, which plays an important role in nitrogen metabolism and animal production. A high diversity of rumen bacterial urease genes was observed in our previous study; however, information on urease protein diversity could not be determined due to technical limitations. Here, we developed a targeted meta-proteomic pipeline to analyze rumen urease protein diversity. Protein extraction (duration of cryomilling in liquid nitrogen), protein digestion state (in-solution or in-gel), and the digestion enzyme used (trypsin or Glu-C/Lys-C) were optimized, and the digested peptides were analyzed by LC-MS/MS. Four minutes was the best duration for cryomilling and yielded the highest urease activity. Trypsin digestion of in-gel proteins outperformed other digestion methods and yielded the greatest number of identifications and superior peptide performance in regards to the digestion efficiency and high-score peptide. The annotation of peptides by PEAKS software revealed diversity among urease proteins, with the predominant proteins being from *Prochlorococcus*, *Helicobacter*, and uncultured bacteria. In conclusion, trypsin digestion of in-gel proteins was the optimal method for the meta-proteomic pipeline analyzing rumen microbial ureases. This pipeline provides a guide for targeted meta-proteomic analyses in other ecosystems.

## Graphical Abstract

Schematic representation of the experimental steps comprised in the targeted meta-proteomic pipeline.

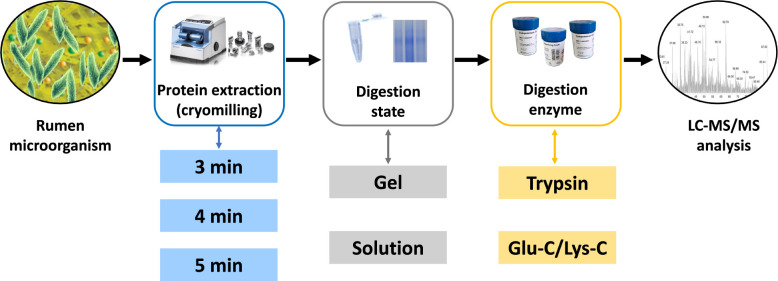

## Introduction

In the diets of ruminants, urea is commonly used as a cost-efficient replacement for animal and vegetable proteins as nitrogen sources (Kertz, 2010; Lin et al., 2012). Urea is also recycled into the rumen through saliva and the ruminal wall. In the rumen, urease produced by ureolytic bacteria catalyzes the hydrolysis of urea into ammonia, which can then be synthesized into microbial proteins to support requirement for the animal growth, along with meat and milk production (Milton et al., 1997; Reynolds and Kristensen, 2008). However, excessive urease activity in the rumen produces high amounts of ammonia that often exceed the rate of ammonia absorption, thus leading to ammonia toxicity in ruminants and environmental nitrogen pollution (Powell et al., 2011; Patra, 2015). In ruminants, ureolytic bacteria and microbial urease are crucial control factors for the improvement of urea-N utilization.

To better understand urea metabolism in the rumen, some ureolytic bacteria were isolated and identified using a culture-dependent method, and different ureolytic bacteria demonstrated varying urease activities (Cook, 1976; Lauková and Koniarová, 1995; Patra, 2018). However, those earlier bacterial isolates are not representative of all ureolytic bacterial strains in the rumen, and it has been estimated that >99% of microorganisms are undetected when obtaining pure cultures (Michael and Giovannoni, 2003). Using high-throughput sequencing of the bacterial 16S rRNA genes, Jin et al. (2016) revealed that rumen ureolytic bacteria were abundant in the genera of *Pseudomonas*, *Haemophilus*, *Neisseria*, *Streptococcus*, *Actinomyces*, *Bacillus*, and unclassified *Succinivibrionaceae*. They also identified a high degree of urease gene sequence diversity by high-throughput sequencing of the urease gene, *ureC*, from ruminant microbial populations. More than 55% of the *ureC* sequences did not affiliate with any known taxonomically assigned urease genes (Jin et al., 2017). Many rumen ureolytic bacteria have been identified using gene sequencing technology, while the actual function of ureolytic bacteria remain unknown. Predictions based on gene sequencing technology have shown considerable redundancy in the rumen ecosystem (Hess et al., 2011; Hart et al., 2018). It is not certain that a given DNA will express an active protein, and the functionality of the protein encoded needs to be further confirmed.

Meta-proteomics has been described as the complete protein complement of microbiota in an environment at a given point in time (Wilmes and Bond, 2004). Thus, meta-proteomics has the potential to provide a more complete description of the activities of the rumen microbiota in this complex ecosystem. Some researchers attempted to study rumen microorganisms using meta-proteomics. Deusch and Seifert (2015) evaluated three different sample preparation approaches for meta-proteomic studies of rumen bacteria using an in-solution digestion method. Snelling and Wallace (2017) suggested that a solution-based digestion method relied on databases in which the great majority of rumen bacterial strains were not represented. Thus, they explored the rumen microbial meta-proteome in bovine and ovine using 2D SDS-PAGE gels. However, to the best of our knowledge, no meta-proteomics study of rumen ureolytic bacteria has been published previously.

The rumen microbial community is composed of thousands of species of micro-organisms, and evaluating the function of the proteome of target bacteria is not a trivial task. This is particularly true when determining the function of key enzymes generated by multiple species. To improve the coverage and depth of protein identification, attempts to refine meta-proteomic pipelines have been reported in other ecosystems (Harris et al., 2007; Oliveira et al., 2014; Tanca et al., 2016), where newly identified proteins were confirmed, and many of which were previously found to be present in very low abundance.

The aim of this study was to develop a targeted meta-proteomic pipeline to analyze rumen urease diversity at the protein level. Protein extractions of high activity ureases were assessed first for subsequent targeted meta-proteomic analyses. Four different digestion methodologies were assessed to evaluate the effects different digestion states (in-solution or in-gel) and the digestion enzymes used (trypsin or Glu-C/Lys-C) on meta-proteomic analyses.

## Materials and Methods

### Protein Preparation

Ruminal digesta samples were collected from three cattle with ruminal cannulas and filtered through four layers of cheesecloth to isolate the ruminal fluid. The mixture of ruminal fluid was immediately divided into centrifuge tubes, placed on dry ice, and transferred to the laboratory where they were stored in liquid nitrogen until analysis. Ruminal fluid samples were thawed with cold running water, centrifuged at low-speed (300 *g* at 4°C for 10 min) to remove large particles and protozoa, and then the supernatants were centrifuged at high-speed (12,000 *g* at 4°C for 5 min) to collect microbial cells. The harvested cells were washed twice by resuspension in cold 50 mM 2-[4-(2-hydroxyethyl)piperazin-1-yl]ethanesulfonic acid (HEPES, pH 7.5) buffer followed by high-speed centrifugation. A 5 mL aliquot was used for protein extraction by cryomilling in liquid nitrogen at a speed of 5/s (Retsch, Haan, Germany). Different cryomilling durations were assessed. The resulting protein samples (supernatants) were collected by centrifugation at 16,000 *g* at 4°C for 10 min. The protein samples were fractionated and preserved in liquid nitrogen pending proteomic analysis. The same samples were used for shotgun assays and gel where 12% SDS-PAGE gel was stained with Coomassie Brilliant Blue.

### Urease Activity Assay

Following protein extraction, protein concentrations were determined using a Bradford Protein Assay Kit (Takara, Dalian, China), according to the manufacturer’s instructions. The extracted proteins were incubated with 100 mM urea at 37°C for 20 min. Phenol-nitroprusside and hypochlorite were then added and, after incubation at 37°C for 30 min, urease activity was determined based on the amount of ammonia released, as described previously (Weatherburn, 1967; Fong et al., 2013). The concentration of ammonia released was measured by a modified phenol/hypochlorite reaction method. One unit of urease activity was defined as 1 nmol of ammonia released per min per mg of microbial protein.

### In-Solution Digestion and In-Gel Digestion

A total of 25 μg of protein was used for in-solution digestions as described previously (Zhang et al., 2016). Protein was first buffer-exchanged into 25 mM of ammonium bicarbonate. Subsequently, the sample was reduced with 1 mM dithiothreitol (DTT) in 25 mM ammonium bicarbonate at 56°C for 30 min, followed by alkylation using 1 mM iodoacetamide (IAA) in 25 mM ammonium bicarbonate, and incubation in the dark for 30 min. Different digestion enzymes were diluted to 0.5 μg/μL with 25 mM ammonium bicarbonate. For trypsin digestion, 1 μL trypsin (Sigma-Aldrich, St. Louis, MO, United States) was added into the reduced and alkylated protein for overnight digestion at 37°C with agitation. For Glu-C/Lys-C digestion, after 2 μL Glu-C digestion (Sigma-Aldrich, St. Louis, MO, United States) at 37°C for 6 h, 2 μL Lys-C (Sigma-Aldrich, St. Louis, MO, United States) was added for overnight digestion. After digestion, the solution was dried down using a centrifugal vacuum concentrator.

For in-gel digestions, gel lane of 50–70 kDa in the molecular weight range of urease were isolated as shown in Supplementary Figure S1. Decolorization of the protein lane was first performed with 200 μL of destaining buffer [50 mM ammonium bicarbonate with acetonitrile (100/100 v/v)] at 37°C for 10 min. Destaining was repeated until decolorization was complete. After one wash with 100 μL acetonitrile, the destained protein lane was reduced and alkylated using 10 mM DTT and 55 mM IAA, respectively. The protein lane was washed again with acetonitrile and then incubated in 50 μL of 25 mM ammonium bicarbonate for 10 min. Different digestion enzymes were added as described for the in-solution digestions. After overnight digestion, the peptides were incubated in 200 μL of extraction buffer (5% trifluoroacetic acid) at 37°C for 1 h, centrifuged (14,000 *g* for 5 min), and the supernatants were transferred into fresh tubes. Samples were then dried and stored at −20°C.

Dried peptides from in-solution and in-gel digestions were dissolved in 0.1% formic acid (FA), desalted using C18 spin columns (Thermo Fisher Scientific, Schwerte, Germany), and eluted using 70% acetonitrile with 0.1% FA. After evaporation, the digested peptides were resuspended in 2% methanol containing 0.1% FA for mass spectrometry analysis on a Q Exactive HF-X instrument (Thermo Fisher Scientific, Schwerte, Germany).

### LC-MS/MS Analysis

The final concentration of resuspended peptides was diluted to about 100 μg/mL, and 10 μL of the peptide mixture was loaded onto a precolumn (Acclaim PepMap100 column, 2 cm × 100 μm, C18, 5 μm) at 3 μL/min for 8 min before being resolved onto a chromatographic column (EASY-Spray column, 12 cm × 75 μm, C18, 3 μm). Peptide separation was carried out at a flow rate of 600 nL/min for 88 min. For peptide ionization, a spray voltage of 2.3 kV was used and a capillary temperature of 350°C was applied. Data-dependent acquisition with full scans in the 350–1,550 m/z range was performed at a resolution of 120,000 FWHM. The targeted automatic gain control (AGC) value for MS1 and MS2 scan modes was set to 3 × e^6^ and 2 × e^4^, respectively. The maximum injection time for full scans and MS/MS scans were 20 and 30 ms, respectively. The normalized collision energy (NCE) was 27%. The mass spectrometry data were stored on the local server for data analysis.

### Data Analysis

Protein identification and data analyses were conducted using PEAKS studio X plus software (Bioinformatics Solutions Inc., Waterloo, Canada) using the standard workflow of *de novo* analysis, the PEAKS DB search, and spider tools. For input parameters, the precursor mass tolerance and fragment mass tolerance were set at 20.0 ppm and 0.05 Da, respectively, with a maximum of three missed cleavages per peptide. The percentages of the average local confidence (ALC) presented the average of the sum of the confidence that a particular amino acid was present in the *de novo* sequences at a particular position (Ngoh and Gan, 2018). To obtain more accurately predicted peptides, we set the ALC for *de novo* peptides as ≥80%. In addition, an in-house database was constructed, containing known urease sequences from the NCBI database and unclassified urease sequences identified by us previously, revealing the targeted meta-proteomic analysis of rumen microbial ureases in this study. The PEAKS peptide score (−10lgP) is derived from the *P*-value that indicates the statistical significance of the peptide-precursor spectrum match. The PEAKS protein score is the weighted sum of the −10lgP peptide scores (Kaddour et al., 2020). The −10lgP of proteins and peptides was set to 15. The identified proteins had at least two unique peptides. All of the identified proteins were further classified as protein groups when they shared at least one identified peptide or an identical peptide set.

### Statistical Analysis

The statistical significance for the differences between two groups was assessed using a *t*-test, and one-way analysis of variance (ANOVA) with a Tukey’s *post hoc* test in OriginPro 9.1 (OriginLab, MA, United States) to compare multiple groups. Statistical significance was accepted at *P* < 0.05.

## Results

### Effects of Cryomilling on Protein Concentration and Urease Activity

**FIGURE 1:**
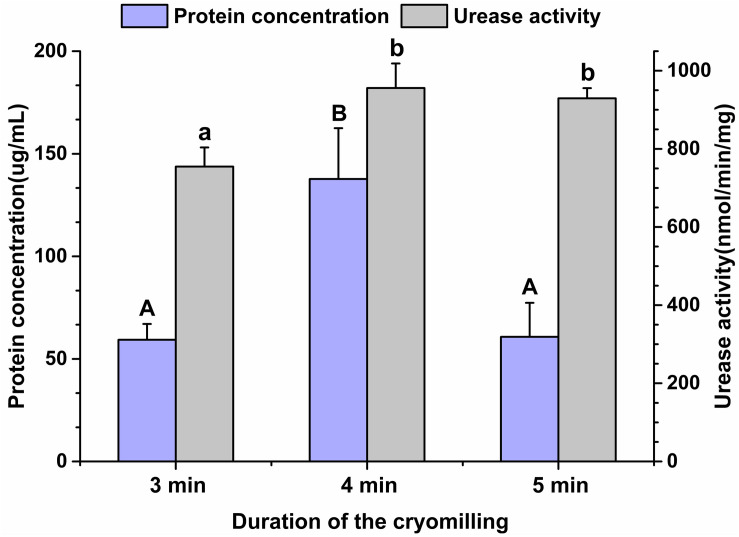
Effects of cryomilling on protein concentration and urease activity. Cryomilling in liquid nitrogen for 3, 4, and 5 min. Significance is indicated as letters (capital letters for groups of protein concentrations and lowercase letters for groups of urease activities). The groups with different letters were significantly different (*P* < 0.05).

Given the high gene diversity of rumen microbial ureases, targeting the regulation of high-activity ureases will be effective for improving urea utilization efficiency and reducing urinary nitrogen excretion in animals. To assess the suitability of the cryomilling method of protein extraction, the effects of cryomilling duration on urease activity and protein concentration were evaluated. The results showed that cryomilling in liquid nitrogen for 4 min maximized urease activity (955.73 nmol/min/mg) and protein concentration (137.73 μg/mL) compared to cryomilling for 3 or 5 min (Figure 1).

### Effects of Different Digestion Methods on Targeted Meta-Proteomic Analyses of Rumen Microbial Urease

Four different digestion methodologies (trypsin digestion of in-solution proteins, trypsin digestion of in-gel proteins, Glu-C/Lys-C digestion of in-solution proteins, and Glu-C/Lys-C digestion of in-gel proteins) were assessed to select the optimum method for targeted meta-proteomic analyses of rumen microbial urease. These approaches focused on assessing different digestion states (in-solution and in-gel) and digestion enzymes used (trypsin and Glu-C/Lys-C), in which tandem Glu-C/Lys-C digestion was a potential replacement for trypsin digestion to perform the greatest number of urease peptides by virtual digestion of different digestion enzymes (Supplementary Figure S2).

**FIGURE 2:**
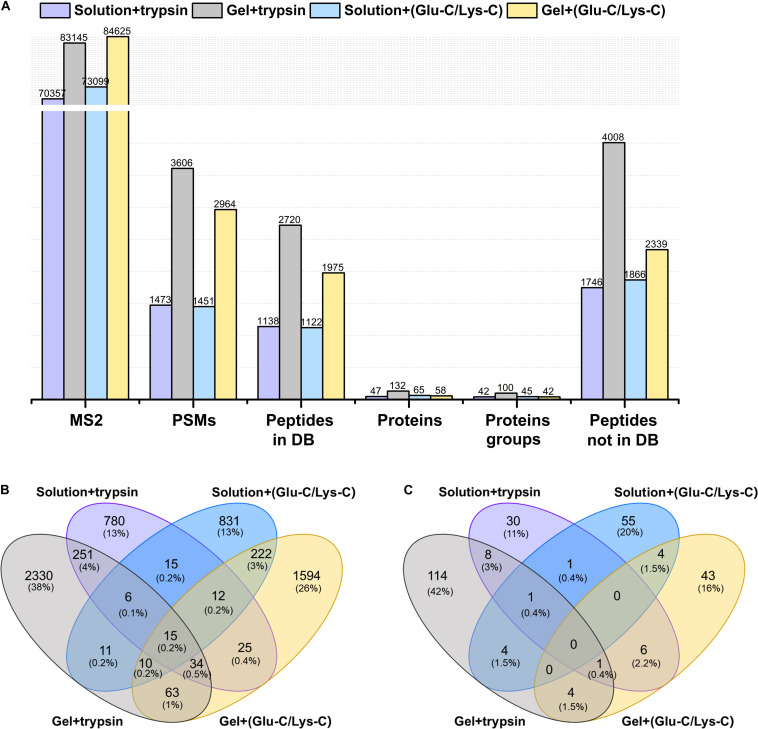
Comparison of different digestion methods in the targeted meta-proteomic analyses of rumen microbial ureases. **(A)** Effects of different digestion methods on identifications. Solution + trypsin, trypsin digestion of in-solution proteins; Gel + trypsin, trypsin digestion of in-gel proteins; solution + (Glu-C/Lys-C), Glu-C/Lys-C digestion of in-solution proteins; Gel + (Glu-C/Lys-C), Glu-C/Lys-C digestion of in-gel proteins; MS2, MS/MS scans; PSMs, peptide spectrum matches; peptides in DB, peptides identified from the database; proteins, identified proteins in the database; proteins groups, all identified proteins were classified as protein groups when they shared at least one identified peptide or an identical peptide set; peptides not in DB, *de novo* only spectra, corresponding to the existing *de novo* sequencing results without peptide identifications in the database. Thus, the peptides were not identified from the database. **(B)** Venn diagram of the peptides identified from the database. **(C)** Venn diagram of identified proteins.

The actual identification results of the four different digestion methodologies were shown in Figure 2A. Trypsin digestion of in-gel protein outperformed other digestion methods, with the greatest number of identifications in peptide spectrum matches (PSMs), peptides from and not from the database, proteins, and protein groups. Such improvement were at least 37.72% more peptides (from the database) and 103.07% more proteins than others. In addition, trypsin digestion of in-gel proteins also yielded the highest number of exclusively identified peptides (from the database) and proteins with 38 and 42%, respectively (Figures 2B,C). For MS/MS scans (MS2), Glu-C/Lys-C digestion exhibited slightly better performance than trypsin digestion, regardless of whether in-solution digestion or in-gel digestion was performed. Those findings were consistent with the results expected based on virtual digestion. Interestingly, for the in-solution digestions, different digestion enzymes had less of an influence on the number of identifications than they did in in-gel digestions.

### Evaluation of Peptide Identifications Using Different Digestion Methods

**FIGURE 3:**
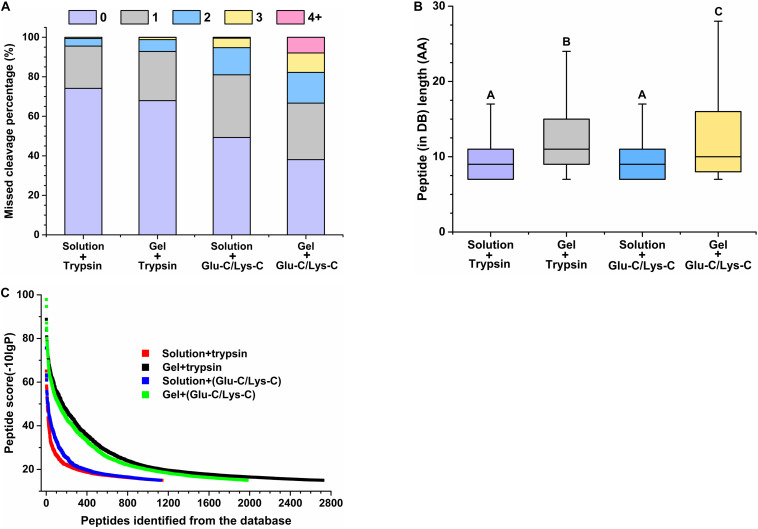
Evaluation of peptide identifications using different digestion methods. **(A)** Percentage of missed cleavage sites. 0–4 represent 0–4 missed cleavage sites. **(B)** Length of identified peptides. Significance is indicated as letters, and the groups with different letters were significantly different (*P* < 0.05). **(C)** Score distribution of identified peptides. Peptides represent peptides identified from the database (DB).

The performance of peptides identified from the database was further assessed by determining the percentage of missed cleavage sites, the lengths of the identified peptides, and the score distribution of the identified peptides. The digestion efficiency of a digestion enzyme is reflected by the number of missed potential cleavage sites within the identified peptides. As shown in Figure 3A, the missed cleavage sites varied from zero to four following in-solution digestions; the zero missed cleavage sites accounted for 74.17% in trypsin digestions, whereas it accounted for only 49.29% in Glu-C/Lys-C digestions. Similar trends were observed in in-gel digestions, indicating that trypsin was a superior digestion enzyme than Glu-C/Lys-C.

Regardless of whether trypsin or Glu-C/Lys-C was used, the average peptide length of in-solution digestions was significantly shorter than that observed with in-gel digestions (*P* < 0.05) (Figure 3B), revealing that the digestion medium was the major influencing factor for the identified peptide length. We further demonstrated that the digestion state was also the main factor influencing the identified peptide score (Figure 3C). Additionally, a greater number of high-score peptides were produced using the in-gel digestion method. Considering the highest number of identifications in peptides and proteins, as well as the superior peptide performance in digestion efficiency and high-score peptides, trypsin digestion of in-gel proteins exhibited the best performance for widespread use in targeted meta-proteomics analyses of rumen microbial urease.

### Diversity of Rumen Microbial Urease

**FIGURE 4:**
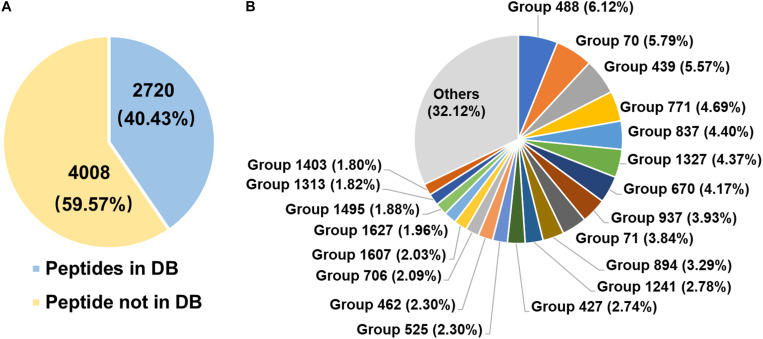
Diversity of rumen microbial ureases. **(A)** Percentage of peptides. Peptides in DB, peptides identified from the database; peptides not in DB, peptides not identified from the database. **(B)** Relative abundance of identified protein groups (TOP 20). Group number, identified protein group ID. Details of identified protein groups were shown in Supplementary Table S1.

The diversity of rumen microbial urease was investigated at the peptide and protein levels using trypsin digestion of in-gel protein method. In total, 2,720 peptides identified from the database, 4,008 peptides not identified from the database, and 132 identified proteins were obtained. The peptide feature area is used for peptide, protein, and protein group abundance calculation. The relative abundance of the identified protein groups and the top 20 protein groups using trypsin digestion of in-gel protein method were shown in Figure 4B and Supplementary Table S1. Approximately 70% of protein groups were attributed to the top 20 abundant, and the changes in abundance among those protein groups were slight. The identified proteins were from *Prochlorococcus marinus*, *Helicobacter heilmannii*, *Thalassobacillus devorans*, *Sporolactobacillus* sp. THM7-4, *Cellulosimicrobium cellulans*, *Sporosarcina psychrophila*, and some unknown bacteria. Those data indicated the protein diversity among rumen microbial ureases. Moreover, at the peptide level, 59.57% peptides were not identified from the database (Figure 4A). Identified proteins from the second most abundant protein groups and many other proteins were matched to urease sequences previously identified by our laboratory, suggesting that there were many unknown microbial ureases in the rumen.

## Discussion

In rumens, microbial urease hydrolyzes urea to ammonia, which is then used for the synthesis of microbial proteins that play an important role in non-protein-related nitrogen metabolism. High urease activities lead to the reduced synthesis of urea into microbial proteins and higher emissions of nitrogen in urine.

The diversity of urease genes in rumen ureolytic bacteria is high. Thus, it is important to identify the predominant urease or ureolytic bacterium, and characterize their functions, which could be beneficial for designing efficient inhibition strategies. Therefore, rumen microbial proteins with high urease activity were extracted first.

As a type of mechanical cell disruption method, bead beating not only facilitated protein extraction and identification (Tanca et al., 2014; Young et al., 2015) but also significantly increased the relative abundances of Firmicutes and Actinobacteria in meta-proteomic analyses of human gut microbiomes (Zhang et al., 2018). Interestingly, Actinomyces have been reported to be a ureolytic bacterium in rumen (Jin et al., 2016). In addition, liquid nitrogen is one of the most commonly used methods for cryogenic sample processing, especially for protein samples, which can effectively retain protein function and activity. Accordingly, cryomilling in liquid nitrogen was performed to extract functional rumen microbial proteins. The extraction of highly active urease was further optimized by comparing different cryomilling durations.

Multi-enzyme digestion has been used to increase proteolytic efficiency, as well as the coverage for comprehensive meta-proteomics analyses. Commonly used digestion enzymes (Zhang et al., 2013) and their combinations were used for virtual digestions of urease proteins. The combination of Glu-C and Lys-C produced the highest number of virtual peptides in the ranges identified by mass spectrometry, while the actual performance was worse. In the in-solution digestion assays, the performances of different enzymes were similar. In the in-gel digestion assays, only at the level of MS2, Glu-C/Lys-C digestion identified slightly more MS2 than trypsin digestion, while at other levels, identifications from Glu-C/Lys-C digestion were lower, which may be related to the lower percentage of zero-missed cleavage sites in Glu-C/Lys-C digestion. Lys-C is the second most widely used protein-digestion enzyme and exhibits efficient cleavage at Lys-X bonds (Jekel et al., 1983), which can make up for the relatively low cleavage at Lys-X bonds for trypsin (Huesgen et al., 2015; Wu et al., 2018). However, some studies have indicated that peptide identifications from Lys-C/trypsin digestions were fewer than those following trypsin digestion alone (Klammer and Maccoss, 2006; Nilsson et al., 2010).

In addition to evaluating the effect of multi-enzyme digestion on the identification of rumen microbial ureases, two commonly used digestion approaches (in-solution and in-gel) in meta-proteomics analyses were also compared. Due to methodological peculiarities, the in-solution digestion method facilitated a rapid scan of nearly the complete proteome, but with less accuracy, while the in-gel digestion approach usually provided a focus on the identification of specific proteins under experimental conditions (Oliveira et al., 2014). Such findings were consistent with the purpose of our study to develop a targeted meta-proteomic pipeline using trypsin digestion of in-gel proteins to analyze rumen urease diversity.

The identifications of PSMs, peptides, proteins, and protein groups by trypsin digestion of in-gel proteins method were 1.45-times, 1.39-times, 1.81-times, and 1.38-times higher, respectively, than that of the trypsin digestion of in-solution proteins method. Thus, those data suggested that trypsin digestion of in-gel proteins was a straightforward and efficient method for targeted meta-proteome analyses. Goldman et al. (2019) reported similar results from the in-solution digestion of 5 μg of cell lysates, which led to the identification of much fewer and more variable proteins compared to that obtained using in-gel digestions.

To improve the protein identifications, attempts to combine different digestion methods have been reported in other ecosystems. Bazile et al. (2019) combined in-gel and in-solution digestions to identify 26 new candidate biomarkers contributing to marbling in bovine using a meta-proteomics approach. Jia et al. (2018) revealed a pre-mRNA splicing factor as a new E7070-dependent DCAF15 substrate using two complementary digestion methods (Lys-C/Trypsin and Lys-N/LysargiNase). In this study, the best-performing trypsin digestion of in-gel proteins method was combined with three other digestions methods (Figures 2B,C), improving the numbers of identified peptides and proteins, and with a low percentage of overlaps. For example, the combination of trypsin digestion of in-gel proteins and trypsin digestion of in-solution proteins showed the overlaps of 306 peptides and 10 proteins, and with 4.8 and 3.8%, respectively, revealing the complementarity between the two methods. However, the actual digestion efficiency of Glu-C/Lys-C enzymes was poor, and digestion with multiple enzymes, or their combinations, was tedious and time-consuming. Based on the practical application study, we chose to apply a single trypsin digestion of in-gel proteins method for the proteomic analysis of rumen microbial ureases.

*Prochlorococcus marinus* was observed at a highest relative abundance with 9% protein sequence coverage following trypsin digestion of the in-gel proteins method. It also had the highest relative abundance using Glu-C/Lys-C digestion of in-gel proteins method, with 7% protein sequence coverage. However, *P. marinus* was not detected following any in-solution digestions. Previous studies of samples from oligotrophic oceans revealed that some *P. marinus* strains exhibited urease activity (McDonagh et al., 2012; Gómez-Baena et al., 2015; Domínguez-Martín et al., 2017). *P. marinus* strain PCC 9511 expressed the smallest urease with a specific urease activity of 94.6 μmol/min/mg (Palinska et al., 2001), which was higher than the total urease activity detected in rumens. Those results indicated that *P. marinus* may be a predominant ureolytic bacterium with high expression and high activities of urease in rumen, exhibiting great potential for use as a target for the functional assessment and inhibitor design of rumen microbial urease.

## Conclusion

We developed a targeted meta-proteomic analyses pipeline by a combination between protein extraction of high urease activity and trypsin digestion of in-gel proteins to analyze rumen urease protein diversity. There was a high diversity among urease proteins, with the predominant proteins being from *Prochlorococcus*, *Helicobacter*, and some uncultured bacteria. This pipeline not only extends our understanding of high activity ureases in cattle rumen but also provides a guide for targeted meta-proteomic analyses in other complex ecosystems.

## Data Availability Statement

All datasets presented in this study are included in the article/Supplementary Material.

## Ethics Statement

The animal study was reviewed and approved by the Animal Welfare & Ethics Committee, Institute of Animal Science, Chinese Academy of Agricultural Sciences.

## Author Contributions

XZ, SZ, and JW designed the experiments. XZ and YH performed the experiments. XZ and SZ analyzed the data and wrote the manuscript. SZ, NZ, and XZ revised the manuscript. SZ, NZ, and JW acquired the funding and supervised the project. All authors agreed to be accountable for all aspects of the work.

## Conflict of Interest

The authors declare that the research was conducted in the absence of any commercial or financial relationships that could be construed as a potential conflict of interest.

## Supplementary Material

FIGURE S1 SDS-PAGE of rumen microbial proteins. Lane M: page ruler prestained protein ladder (Thermo Fisher Scientific, Schwerte, Germany). Lane 1, lane 2, and lane 3: Different amounts of rumen microbial proteins. Protein lanes in the orange box were isolated for in-gel trypsin digestion, while protein lanes in the red box were isolated for in-gel Glu-C/Lys-C digestion.

FIGURE S2 Effects of different virtual digestions on peptide length (AA). Urease protein sequences from the NCBI database were digested by different digestion enzymes using Peptide Mass software (https://web.expasy.org/peptide_mass/).

TABLE S1 Identified urease proteins in top 20 protein groups. Accession is the accession number of a protein in NCBI database. ^∗^ was unclassified urease sequence identified by us previously.

## References

[B1] Bazile J. Picard B. Chambon C. Valais A. Bonnet M. (2019). Pathways and biomarkers of marbling and carcass fat deposition in bovine revealed by a combination of gel-based and gel-free proteomic analyses. *Meat Sci.* 156 146–155. 10.1016/j.meatsci.2019.05.018 31158601

[B2] Cook A. R. (1976). The elimination of urease activity in Streptococcus faecium as evidence for plasmid coded urease. *J. Gen. Microbiol.* 9 49–58. 10.1099/00221287-92-1-49 1107485

[B3] Deusch S. Seifert J. (2015). Catching the tip of the iceberg – evaluation of sample preparation protocols for the metaproteomic studies of the rumen microbiota. *Proteomics* 15 3590–3595. 10.1002/pmic.201400556 25765363

[B4] Domínguez-Martín M. A. Gómez-Baena G. Díez J. López-Grueso M. J. Beynon R. J. García-Fernández J. M. (2017). Quantitative proteomics shows extensive remodeling induced by nitrogen limitation in *Prochlorococcus marinus* SS120. *mSystems* 2:e00008-17. 10.1128/mSystems.00008-17 28593196 PMC5451487

[B5] Fong Y. H. Wong H. C. Yuen M. H. Lau P. H. Chen Y. W. Wong K. B. (2013). Structure of UreG/UreF/UreH complex reveals how urease accessory proteins facilitate maturation of *Helicobacter pylori* urease. *PLoS Biol.* 11:e1001678. 10.1371/journal.pbio.1001678 24115911 PMC3792862

[B6] Goldman A. R. Beer L. A. Tang H. Y. Hembach P. Zayas-Bazan D. Speicher D. W. (2019). Proteome analysis using gel-LC-MS/MS. *Curr. Protoc. Protein Sci.* 96:e93. 10.1002/cpps.93 31180188 PMC6653605

[B7] Gómez-Baena G. Domínguez-Martín M. A. Donaldson R. P. García-Fernández M. Diez J. (2015). Glutamine synthetase sensitivity to oxidative modification during nutrient starvation in *Prochlorococcus marinus* PCC 9511. *PLoS One* 10:e0135322. 10.1371/journal.pone.0135322 26270653 PMC4535847

[B8] Harris L. R. Churchward M. A. Butt R. H. Coorssen J. R. (2007). Assessing detection methods for gel-based proteomic analyses. *J. Proteome Res.* 6 1418–1425. 10.1021/pr0700246 17367184

[B9] Hart E. H. Creevey C. J. Hitch T. Kingston-Smith A. H. (2018). Meta-proteomics of rumen microbiota indicates niche compartmentalisation and functional dominance in a limited number of metabolic pathways between abundant bacteria. *Sci. Rep.* 8:10504. 10.1038/s41598-018-28827-7 30002438 PMC6043501

[B10] Hess M. Sczyrba A. Egan R. Kim T. W. Chokhawala H. Chroth G. (2011). Metagenomic discovery of biomass-degrading genes and genomes from cow rumen. *Science* 331 463–467. 10.1126/science.1200387 21273488

[B11] Huesgen P. F. Lange P. F. Rogers L. D. Solis N. Eckhard U. Kleifeld O. (2015). LysargiNase mirrors trypsin for protein C-terminal and methylation-site identification. *Nat. Methods* 12 55–58. 10.1038/nmeth.3177 25419962

[B12] Jekel P. A. Weijer W. J. Beintema J. J. (1983). Use of endoproteinase Lys-C from Lysobacter enzymogenes in protein sequence analysis. *Anal. Biochem.* 134 347–354. 10.1016/0003-2697(83)90308-1 6359954

[B13] Jia X. Pan L. Zhu M. Hu H. Zhai L. Liu J. (2018). pSILAC method coupled with two complementary digestion approaches reveals PRPF39 as a new E7070-dependent DCAF15 substrate. *J. Proteomics* 210:103545. 10.1016/j.jprot.2019.103545 31626998

[B14] Jin D. Zhao S. Wang P. Zheng N. Bu D. Beckers Y. (2016). Insights into abundant rumen ureolytic bacterial community using rumen simulation system. *Front. Microbiol.* 7:1006. 10.3389/fmicb.2016.01006 27446045 PMC4923134

[B15] Jin D. Zhao S. Zheng N. Bu D. Beckers Y. Denman S. E. (2017). Differences in ureolytic bacterial composition between the rumen digesta and rumen wall based on ureC gene classification. *Front. Microbiol.* 8:385. 10.3389/fmicb.2017.00385 28326079 PMC5339240

[B16] Kaddour H. Lyu Y. Welch J. L. Paromov V. Mandape S. N. Sakhare S. S. (2020). Proteomics profiling of autologous blood and semen exosomes from HIV-infected and uninfected individuals reveals compositional and functional variabilities. *Mol. Cell Proteomics* 19 78–100. 10.1074/mcp.RA119.001594 31676584 PMC6944229

[B17] Kertz A. F. (2010). Review: urea feeding to dairy cattle: a historical perspective and review. *Prof. Anim. Sci.* 26 257–272. 10.15232/s1080-7446(15)30593-3

[B18] Klammer A. A. Maccoss M. J. (2006). Effects of modified digestion schemes on the identification of proteins from complex mixtures. *J. Proteome Res.* 5 695–700. 10.1021/pr050315j 16512685 PMC2535816

[B19] Lauková A. Koniarová I. (1995). Survey of urease activity in ruminal bacteria isolated from domestic and wild ruminants. *Microbios* 84 7–11. 8569526

[B20] Lin W. Mathys V. Ang E. L. Y. Koh V. H. Q. Gómez J. M. M. Ang M. L. T. (2012). Urease activity represents an alternative pathway for Mycobacterium tuberculosis nitrogen metabolism. *Infect. Immun.* 80 2771–2779. 10.1128/IAI.06195-11 22645285 PMC3434571

[B21] McDonagh B. Domínguez-Martín M. A. Gómez-Baena G. López-Lozano A. Diez J. Bárcena J. A. (2012). Nitrogen starvation induces extensive changes in the redox proteome of *Prochlorococcus* sp. strain SS120. *Environ. Microbiol. Rep.* 4 257–267. 10.1111/j.1758-2229.2012.00329.x 23757281

[B22] Michael S. R. Giovannoni S. J. (2003). The uncultured microbial majority. *Annu. Rev. Microbiol.* 57 369–394. 10.1146/annurev.micro.57.030502.090759 14527284

[B23] Milton C. T. Brandt R. T. Titgemeyer E. C. (1997). Urea in dry-rolled corn diets: finishing steer performance, nutrient digestion, and microbial protein production. *J. Anim. Sci.* 75 1415–1424. 10.2527/1997.7551415x 9159292

[B24] Ngoh Y. Y. Gan C. Y. (2018). Identification of Pinto bean peptides with inhibitory effects on α-amylase and angiotensin converting enzyme ACE activities using an integrated bioinformatics-assisted approach. *Food Chem.* 267 124–131. 10.1016/j.foodchem 29934146

[B25] Nilsson T. Mann M. Aebersold R. Yates J. R. Bairoch A. Bergeron J. J. M. (2010). Mass spectrometry in high-throughput proteomics: ready for the big time. *Nat. Methods* 7 681–685. 10.1038/nmeth0910-681 20805795

[B26] Oliveira B. M. Coorssen J. R. Martins-de-Souza D. (2014). 2DE: the phoenix of proteomics. *J. Proteomics* 104 140–150. 10.1186/s40168-016-0196-8 24704856

[B27] Palinska K. A. Jahns T. Rippka R. Marsac N. T. (2001). Prochlorococcus marinus strain PCC 9511, a *Picoplanktonic cyanobacterium*, synthesizes the smallest urease. *Microbiology* 146(Pt 12), 3099–3107. 10.1099/00221287-146-12-3099 11101668

[B28] Patra A. K. (2015). “Urea/Ammonia metabolism in the rumen and toxicity in ruminants,” in *Rumen Microbiology*, eds Puniya A. Singh R. Kamra D. (Berlin: Springer), 329–341. 10.1007/978-81-322-2401-3_22

[B29] Patra A. K. (2018). Aschenbach, J.R. Ureases in the gastrointestinal tracts of ruminant and monogastric animals and their implication in urea-N/ammonia metabolism: a review. *J. Adv. Res.* 13 39–50. 10.1016/j.jare.2018.02.005 30094081 PMC6077136

[B30] Powell J. M. Wattiaux M. A. Broderick G. A. (2011). Short communication: evaluation of milk urea nitrogen as a management tool to reduce ammonia emissions from dairy farms. *J. Dairy Sci.* 94 4690–4694. 10.3168/jds.2011-4476 21854942

[B31] Reynolds C. K. Kristensen N. B. (2008). Nitrogen recycling through the gut and the nitrogen economy of ruminants: an asynchronous symbiosis. *J. Anim Sci.* 86 E293–E305. 10.2527/jas.2007-0475 17940161

[B32] Snelling T. J. Wallace R. J. (2017). The rumen microbial metaproteome as revealed by SDS-PAGE. *BMC Microbiol.* 17:9. 10.1186/s12866-016-0917-y 28061817 PMC5219685

[B33] Tanca A. Palomba A. Fraumene C. Pagnozzi D. Manghina V. Deligios M. (2016). The impact of sequence database choice on metaproteomic results in gut microbiota studies. *Microbiome* 4:51. 10.1186/s40168-016-0196-8 PMC5037606 27671352

[B34] Tanca A. Palomba A. Pisanu S. Deligios M. Fraumene C. Manghina V. (2014). A straightforward and efficient analytical pipeline for metaproteome characterization. *Microbiome* 2:49. 10.1186/s40168-014-0049-2 25516796 PMC4266899

[B35] Weatherburn M. (1967). Phenol-hypochlorite reaction for determination of ammonia. *Anal. Chem.* 39 971–973. 10.1021/ac60252a045

[B36] Wilmes P. Bond P. L. (2004). The application of two-dimensional polyacrylamide gel electrophoresis and downstream analyses to a mixed community of prokaryotic microorganisms. *Environ. Microbiol.* 6 911–920. 10.1111/j.1462-2920.2004.00687.x 15305916

[B37] Wu Z. Huang J. Li Q. Zhang X. (2018). Lys-C/Arg-C, a more specific and efficient digestion approach for proteomics studies. *Anal. Chem.* 90 9700–9707. 10.1021/acs.analchem.8b02448 30024741

[B38] Young J. C. Pan C. Adams R. M. Brooks B. Banfield J. F. Morowitz M. J. (2015). Metaproteomics reveals functional shifts in microbial and human proteins during a preterm infant gut colonization case. *Proteomics* 15 3463–3473. 10.1002/pmic.201400563 26077811 PMC4607655

[B39] Zhang X. Li L. Mayne J. Ning Z. Stintzi A. Figeys D. (2018). Assessing the impact of protein extraction methods for human gut metaproteomics. *J. Proteomics* 180 120–127. 10.1016/j.jprot.2017.07.001 28705725

[B40] Zhang X. Ning Z. Mayne J. Moore J. I. Li J. Butcher J. (2016). MetaPro-IQ: a universal metaproteomic approach to studying human and mouse gut microbiota. *Microbiome* 4:31. 10.1186/s40168-016-0176-z 27343061 PMC4919841

[B41] Zhang Y. Fonslow B. R. Shan B. Baek M. C. Yates J. R. (2013). Protein analysis by shotgun/bottom-up proteomics. *Chem. Rev.* 113 2343–2394. 10.1021/cr3003533 23438204 PMC3751594

