# Supplementary material for: Pipeline for Targeted Meta-Proteomic Analyses to Assess the Diversity of Cattle Rumen Microbial Urease
Source: Front Microbiol. 2020 Sep 18;11:573414. doi: 10.3389/fmicb.2020.573414 (PMC7531017; doi:10.3389/fmicb.2020.573414)
Supplement: FIGURE S1 — SDS-PAGE of rumen microbial proteins. Lane M: page ruler prestained protein ladder (Thermo Fisher Scientific, Schwerte, Germany). Lane 1, lane 2, and lane 3: Different amounts of rumen microbial proteins. Protein lanes in the orange box were isolated for in-gel trypsin digestion, while protein lanes in the red box were isolated for in-gel Glu-C/Lys-C digestion. [file Data_Sheet_1.docx]

Supplementary Material

## Supplementary Figures


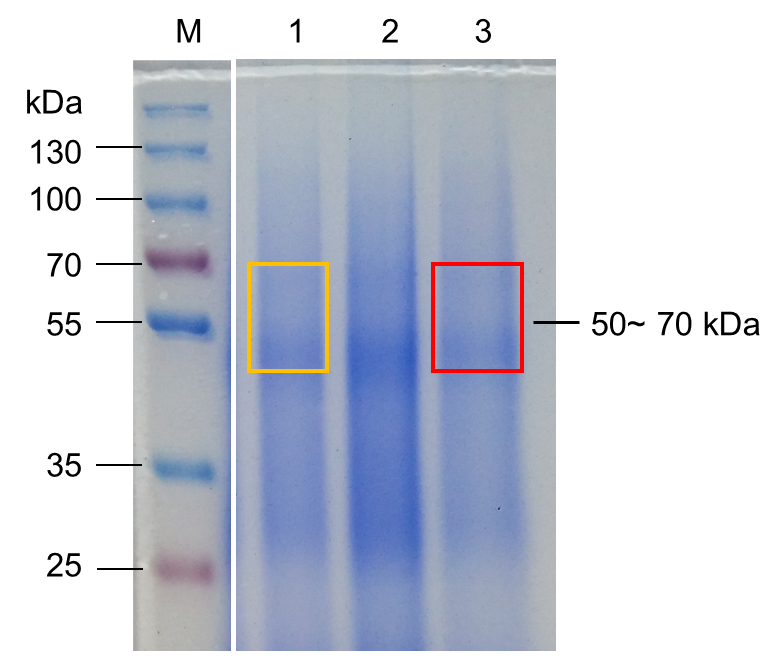


**FIGURE S1 |** SDS-PAGE of rumen microbial proteins. Lane M: page ruler prestained protein ladder (Thermo Fisher Scientific, Schwerte, Germany). Lane 1, lane 2, and lane 3: Different amounts of rumen microbial proteins. Protein lanes in the orange box were isolated for in-gel trypsin digestion, while protein lanes in the red box were isolated for in-gel Glu-C/Lys-C digestion





**FIGURE S2 |** Effects of different virtual digestions on peptide length (AA). Urease protein sequences from the NCBI database were digested by different digestion enzymes using Peptide Mass software (https://web.expasy.org/peptide_mass/).

## Supplementary Table

**TABLE S1 |** Identified urease proteins in top 20 protein groups. Accession is the accession number of a protein in NCBI database. * was unclassified urease sequence identified by us previously.

| **TOP 20** | **Protein group ID** | | **Accession** | **-10lgP** | **Coverage (%)** | **Area** | **Unique peptides** | **Avg. Mass (Da)** | **Description [Taxonomy]** |  |
| --- | --- | --- | --- | --- | --- | --- | --- | --- | --- | --- |
| TOP 1 | | 488 | WP_032513687.1 | 49.46 | 9 | 1.90E+07 | 2 | 61824 | Urease subunit alpha  [*Prochlorococcus marinus*] |  |
| TOP 2 | | 70 | L2Ure_CC_60_C02.exp* | 97 | 11 | 1.80E+07 | 3 | 23661 | Urease subunit alpha  [unclassified bacteria] |  |
|  | | 70 | L2Ure_CC_121_H09.exp* | 97 | 11 | 1.80E+07 | 3 | 23642 | Urease subunit alpha  [unclassified bacteria] |  |
|  | | 70 | L2CC_94_E06* | 97 | 19 | 1.80E+07 | 3 | 13248 | Urease subunit alpha  [unclassified bacteria] |  |
|  | | 70 | L2CC_71_F03* | 97 | 19 | 1.80E+07 | 3 | 13331 | Urease subunit alpha  [unclassified bacteria] |  |
|  | | 70 | L2_CC_45_E06.exp* | 97 | 19 | 1.80E+07 | 3 | 13301 | Urease subunit alpha  [unclassified bacteria] |  |
|  | | 70 | L2_CC_40_H05.exp* | 97 | 19 | 1.80E+07 | 3 | 13216 | Urease subunit alpha  [unclassified bacteria] |  |
| TOP 3 | | 439 | WP_104691060.1 | 50.84 | 8 | 1.73E+07 | 3 | 62012 | Urease subunit alpha  [*Helicobacter heilmannii*] |  |
|  | | 439 | WP_053830447.1 | 50.84 | 8 | 1.73E+07 | 3 | 62045 | Urease subunit alpha  [*Helicobacter heilmannii*] |  |
|  | | 439 | WP_053829597.1 | 50.84 | 8 | 1.73E+07 | 3 | 62027 | Urease subunit alpha  [*Helicobacter heilmannii*] |  |
|  | | 439 | WP_053828936.1 | 50.84 | 8 | 1.73E+07 | 3 | 62027 | Urease subunit alpha  [*Helicobacter heilmannii*] |  |
|  | | 439 | WP_053827914.1 | 50.84 | 8 | 1.73E+07 | 3 | 62043 | Urease subunit alpha  [*Helicobacter heilmannii*] |  |
| TOP 4 | | 771 | WP_028782270.1 | 45.97 | 6 | 1.46E+07 | 2 | 61512 | Urease subunit alpha  [*Thalassobacillus devorans*] |  |
| TOP 5 | | 837 | WP_130032667.1 | 44.42 | 6 | 1.37E+07 | 2 | 60693 | Urease subunit alpha  [*Sporolactobacillus* sp. THM7-4] |  |
| TOP 6 | | 1327 | WP_141389998.1 | 33.52 | 10 | 1.36E+07 | 2 | 59957 | Urease subunit alpha  [*Cellulosimicrobium cellulans*] |  |
|  | | 1327 | WP_064315197.1 | 33.52 | 10 | 1.36E+07 | 2 | 59878 | Urease subunit alpha  [*Cellulosimicrobium* sp. I38E] |  |
|  | | 1327 | WP_047232402.1 | 33.52 | 10 | 1.36E+07 | 2 | 59806 | Urease subunit alpha  [*Cellulosimicrobium funkei*] |  |
| TOP 7 | | 670 | WP_067204306.1 | 41.92 | 6 | 1.30E+07 | 2 | 61397 | Urease subunit alpha  [*Sporosarcina psychrophile*] |  |
| TOP 8 | | 937 | WP_089035260.1 | 30.13 | 4 | 1.22E+07 | 2 | 73446 | Urease subunit alpha  [*Neisseria chenwenguii*] |  |
| TOP 9 | | 71 | WP_112696386.1 | 81.49 | 15 | 1.20E+07 | 2 | 60380 | Urease subunit alpha  [*Rhizobiales bacterium*] |  |
|  | | 71 | WP_112628239.1 | 81.49 | 15 | 1.20E+07 | 2 | 60392 | Urease subunit alpha  [*Rhizobiales bacterium*] |  |
|  | | 71 | WP_109857019.1 | 81.49 | 15 | 1.20E+07 | 2 | 60392 | Urease subunit alpha  [*Ensifer adhaerens*] |  |
| TOP 10 | | 894 | WP_017095202.1 | 43.51 | 6 | 1.03E+07 | 3 | 63934 | Amidohydrolase family protein, urease  [*Vibrio splendidus*] |  |
| TOP 11 | | 1241 | WP_077291490.1 | 36.46 | 4 | 8.66E+06 | 3 | 64746 | Amidohydrolase family protein, urease  [*Labrenzia aggregata*] |  |
| TOP 12 | | 427 | WP_131870262.1 | 57.19 | 11 | 8.51E+06 | 2 | 60607 | Urease subunit alpha  [unclassified *Bradyrhizobium*] |  |
|  | | 427 | WP_042336828.1 | 57.19 | 11 | 8.51E+06 | 2 | 60626 | Urease subunit alpha  [*Bradyrhizobium* sp. DOA9] |  |
| TOP 13 | | 525 | WP_085835677.1 | 46.13 | 8 | 7.17E+06 | 2 | 60619 | Urease subunit alpha  [*Aquimixticola soesokkakensis*] |  |
| TOP 14 | | 462 | WP_106702746.1 | 41.4 | 6 | 7.17E+06 | 2 | 62002 | Urease subunit alpha  [*Ottowia oryzae*] |  |
| TOP 15 | | 706 | WP_139373841.1 | 50.44 | 7 | 6.52E+06 | 2 | 73062 | Urease subunit alpha  [*Enhydrobacter aerosaccus*] |  |
| TOP 16 | | 1607 | WP_136176513.1 | 23.92 | 5 | 6.32E+06 | 2 | 48582 | Amidohydrolase family protein, urease  [*Sphingopyxis* sp. PAMC25046] |  |
| TOP 17 | | 1627 | WP_026439559.1 | 30.5 | 3 | 6.09E+06 | 2 | 60480 | Urease subunit alpha  [*Acidocella facilis*] |  |
|  | | 1627 | WP_008493318.1 | 30.5 | 3 | 6.09E+06 | 2 | 60419 | Urease subunit alpha  [*Acidocella* sp. MX-AZ02] |  |
| TOP 18 | | 1495 | WP_069965646.1 | 28.03 | 3 | 5.86E+06 | 2 | 61375 | Urease subunit alpha  [*Desertifilum* sp. IPPAS B-1220] |  |
| TOP 19 | | 1313 | WP_103715403.1 | 38.27 | 4 | 5.65E+06 | 2 | 61470 | Amidohydrolase family protein, urease  [*Flavobacterium columnare*] |  |
|  | | 1313 | WP_088398703.1 | 38.27 | 4 | 5.65E+06 | 2 | 61412 | Amidohydrolase family protein, urease  [*Flavobacterium columnare*] |  |
| TOP 20 | | 1403 | WP_012863103.1 | 27.48 | 5 | 5.59E+06 | 2 | 50782 | Urease subunit alpha  [*Sebaldella termitidis*] |  |
